# Observed and Normative Discount Functions in Addiction and other Diseases

**DOI:** 10.3389/fphar.2017.00416

**Published:** 2017-06-29

**Authors:** Salvador Cruz Rambaud, María J. Muñoz Torrecillas, Taiki Takahashi

**Affiliations:** ^1^Department of Economics and Business, University of AlmeríaAlmería, Spain; ^2^Department of Behavioral Science, Faculty of Letters, Hokkaido UniversitySapporo, Japan

**Keywords:** delay discounting, addiction, disease, hyperbolic discounting, (generalized) exponentiated hyperbolic discounting, hazard rate

## Abstract

The aim of this paper is to find a suitable discount function able to describe the progression of a certain addiction or disease under treatment as a discounting process. In effect, a certain indicator related to a disease decays over time in a manner which is mathematically similar to the way in which discounting has been modeled. We analyze the discount functions observed in experiments which study addictive and other problematic behaviors as well as some alternative hyperbola-like discount functions in order to fit the patience exhibited by the subject after receiving the treatment. Additionally, it has been experimentally found that people with addiction display high rates of discount (impatience) and preference reversals (dynamic inconsistency). This excessive discounting must be correctly modeled by a suitable discount function, otherwise, it can become a trans-disease process underlying addiction and other disorders. The (generalized) exponentiated hyperbolic discount function is proposed to describe the progression of a disease with respect to the treatment, since it maintains the property of inconsistency by exhibiting a decreasing discount rate after an initial period in which the opposite occurs.

## 1. Introduction

In a recent paper, Bickel et al. (2012) state that drug-dependent individuals, and people with other diseases such as obesity, gambling problems, diagnosed ADHD (Attention Deficit Hiperactivity Disorder) or schizophrenia, discount delayed reinforcers more rapidly than individuals not having these addictions or diseases. It could be said that these individuals are more impulsive or impatient than individuals belonging to the control group (people not suffering from the studied addiction or disease). After an extensive review of the literature on addictive behavior and discounting of delayed rewards, a similar conclusion is drawn by MacKillop et al. (2011) who found strong evidence of greater discounting in individuals with addictive behavior. On the other hand, there are empirical studies in which only (mainly hypothetical) monetary rewards are discounted. Most of these experiments can be found in MacKillop et al. (2011). However, there are also empirical works which compare the discounting of monetary and non-monetary rewards by people with and without addictive behavior. Hypothetical amounts of cigarettes (e.g., Bickel et al., 1999), heroin (e.g., Madden et al., 1997), crack/cocaine (e.g., Coffey et al., 2003), and alcohol (e.g., Petry, 2002) have been used as non-monetary rewards. As a general conclusion, all these substances of abuse were discounted more steeply than money by their consumers. Furthermore, a higher discount was applied to monetary rewards by the group of substance abuse consumers.

According to Prelec (2004), “the core meaning of impatience is a preference for something to happen sooner rather than later.” Some papers (Takahashi et al., 2007) use the term impulsivity as a synonym for impatience^1^. Although Bickel and Marsch (2001) broadly call them “personality assessments,” we will refer to impulsivity in the context of delay discounting.

Another interesting idea, proposed by Bickel et al. (2012), is the consideration of excessive discounting as a trans-disease process underlying addiction and other disorders as well as disease-related behavior. For example, high discount rates appear in smokers and ADHD. This was also pointed out by Green and Myerson (2004) who stated: “Such findings raise the possibility that differences in impulsivity may underlie these behavioral problems^2^ and that assessment based on behavioral discounting measures may be able to predict who is at risk and who is most likely to benefit from interventions.” In a similar fashion, Bickel et al. (2012) propose that “[…] understanding the commonalities in comorbid disorders may inform treatment approaches for multiple disorders.” They also suggest that the relation between disorders and discount rates may be additive and, for example, individuals with two or more disorders could show higher discount rates than individuals with only one disorder. In the same vein, Petry and Casarella (1999) found that the discount rates displayed by people with two disorders, namely gambling and substance abuse, were three times higher than the rates of non-gambling substance abusers. As these disorders additively affected discount rates, we are going to consider an aggregated discount function in this work which takes into account higher discount rates due to more than one disease or addiction. In summary, we will take into account that people with addiction:

1. Have high rates of discount (Bickel et al., 2012, refer to it as excessive rates of discount).
2. Have preference reversals. “These preference reversals are a hallmark feature of individuals suffering from addiction, as they often express a desire to abstain when drugs are not immediately available, but may reverse this preference when the opportunity to use is more proximal […]” (Bickel et al., 2012).
3. Could have a propensity to developing other diseases in which high discount rates are an underlying process.

Indeed, the aforementioned characteristics will help us to find a suitable discount function able to describe these addiction situations. To this end, this paper is organized as follows: In Section 2 we are going to describe a disease or an addiction as a discounting process. Following that, in Section 3, we will first study the discount functions that best fit the behavior of the diseases and their properties. Then, in Section 4, we will analyze some properties of time discounting which may be helpful in the design of the disease therapy. Section 5 proposes excessive discounting as a trans-disease process underlying addiction and other disorders, and explains it through the concept of hazard rate. Finally, Section 6 provides a summary and conclusion.

## 2. Addiction and illnesses as discounting processes

Usually, the term *discounting* is applied to contexts involving delay (delay discounting), or probability (probability discounting). However, several kinds of quantifiable processes may also be viewed as discounting phenomena (Rachlin, 2006). Table 1 shows these processes and introduces the behavioral and psychopharmacological treatment of addictions and diseases which can be considered discounting processes as well. The variable to be discounted (i.e., the original quantity), the discounted quantity and the variable involved in the discounting process is specified for every discounting process in this table. We can distinguish between temporal and non-temporal variables involved in the discounting. In this regard, delay discounting and memory exhibit temporal variables involved in the discounting process, namely, delay to reward and time between learning and recall, respectively. On the other hand, an example of a non-temporal discounting variable would be the social distance from a person in the case of discounting “generosity.” In their experiment, Jones and Rachlin (2006) applied a social discounting equation from Rachlin and Raineri (1992) which included a parameter measuring the social distance (from 1 to 100) between the participants and the person who would be sharing the money, 1 being the closest. The participants had to decide whether to forgo a hypothetical amount of money for themselves in order to give $75 to another person and the amount of money forgone varied with the perceived social closeness to the beneficiary.

**Table 1:** Different types of discounting processes.

**Application of discounting to**	**Original quantity** **Discounted quantity** **Involved variable**
Delay of reward	Absolute reward value
	Current value
	Delay to reward
Probability of reward	Absolute reward value
	Value of probabilistic reward
	Probability of reward
Generosity	Money you have
	Money you give to another person
	Social distance from that person
Energy	Source of energy (e.g., light, sound)
	Energy distant from source
	Distance from source
Memory	Original learning
	Memory
	Time between learning and recall
Addiction or disease	Level of addiction
	Current level
	Regular dose/time frequency

*Source: Rachlin (2006) and own elaboration*.

**Chart 1:**
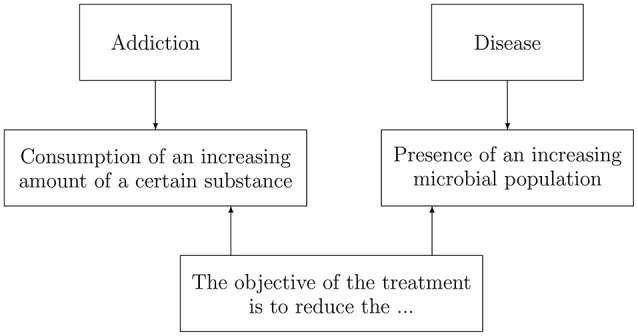
Parallelism between addictions and diseases. Source: own elaboration.

As indicated, in Table 1 we have introduced another process, the behavioral and psychopharmacological treatment of addictions and diseases, which can be considered as a discounting process. In actual fact, all addictions involve the involuntary consumption of an increasing amount of a certain substance. For example, cigarettes (smokers), drugs (drug addicts), and money (compulsive gamblers), among others. The level of an addiction can be defined as the amount of substance which can be consumed in a given period of time (for example, a day). Analogously, for a disease we can assume that an individual is affected by a microbial population. This justifies a joint consideration of addictions and diseases (see Chart 1).

It is well known that many diseases cannot be completely cured. In that situation, treatment attempts to improve or neutralize the problem, especially in chronic diseases. However, this paper is about the subset of treatments which reverse diseases completely or end medical problems permanently. More specifically, we will analyze the diseases treated with a regular dose able to reduce the concentration of pathogens in the patient.

## 3. The discount functions fitting illness or addiction processes

**Figure 1:**
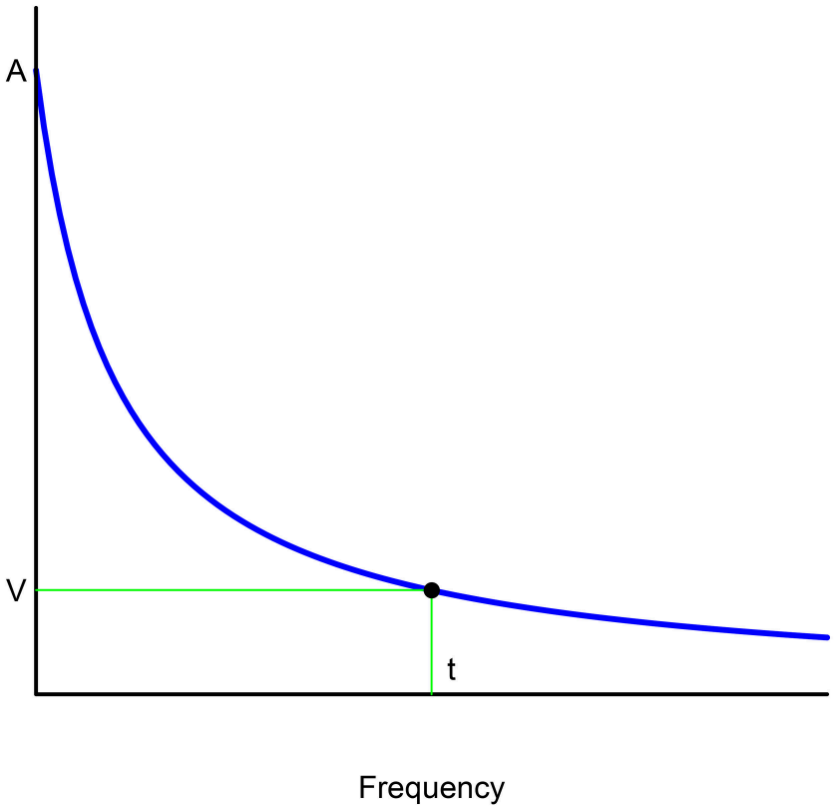
Hyperbolic discounting.

Many experimental studies have shown that the monetary discounting exhibited by people with certain addictions or diseases is best fitted to a hyperbolic discount function (see Figure 1). Accordingly, MacKillop et al. (2011) offer an extensive review of the literature on addictive behavior (addiction to alcohol, tobacco, and gambling, among others) related to discounting of delayed rewards. From the 64 experimental comparisons^3^ reviewed by MacKillop et al. (2011), 70% used Mazur's (1987) hyperbolic discount function. In effect, a simple hyperbola-like is used in the majority of cases to describe the discount in this framework:

(1) $$V=\frac{A}{1+it},$$

where *i* > 0 is a constant discount rate, and *t* is the interval between *A* (the value of the reward to be discounted for *t* periods) and *V* (the value of the reward at instant 0).

Our paper is also in line with the work by Augenblick et al. (2015) who suggest that individuals exhibit hyperbolic discounting for non-monetary goods, but exponential discounting for money. More specifically, the decay in effort is exponential, not hyperbolic, in the delay. This finding is consistent with the evidence of non-present bias on monetary payments (Andreoni and Sprenger, 2012), as opposed to real effort (DellaVigna and Pope, 2017).

According to Bernheim and Rangel (2005), addicts are sometimes allowed to consume involuntarily. To all intents and purposes, this paper introduces five important patterns of addictive behavior: (1) unsuccessful attempts to quit, (2) cue-triggered recidivism, (3) self-described mistakes, (4) self-control through precommitment, and (5) self-control through behavioral and cognitive therapy. Our paper lies in the context of this last group with the implementation of “successful behavioral therapies” able to “teach cue-avoidance, often by encouraging the adoption of new lifestyles and the development of new interests,” as opposed to item 4 where addicts exhibit a “tendency to make mistakes by voluntarily removing or degrading future options.”

Story et al. (2014) assimilate the progression of an illness to a discounting process when stating: “Individuals who are willing to accept a more severe illness occurring after a delay over a less severe immediate illness are said to discount future illness.” According to Ganiats et al. (2000), health evolution can be described as an improvement in health from an initial state of illness, where individuals prefer immediate over delayed health improvement. More specifically, the discounting process is hyperbolic in the way that individuals are willing to accept a smaller-sooner improvement in health over a larger-later improvement. Going beyond this idea, “high discount rates for money (and in some instances for food or drug rewards) are associated with several unhealthy behaviors and markers of health status, establishing discounting as a promising predictive measure” (Story et al., 2014).

**Figure 2:**
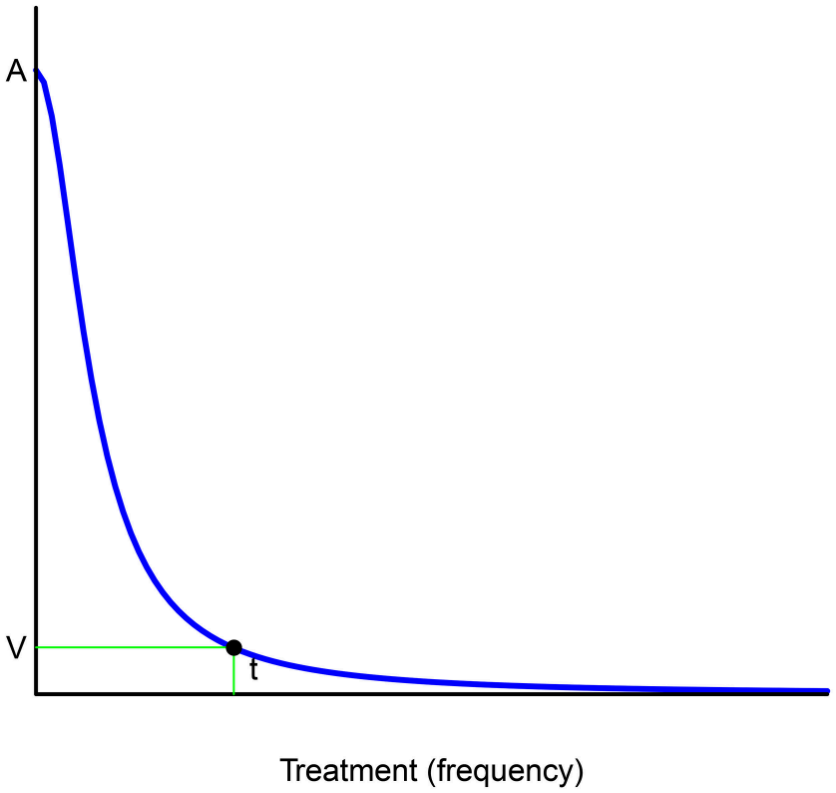
Exponentiated hyperbolic discounting.

This section provides an approach toward justifying the exponentiated hyperbolic discount function as a new discounting model better able to describe the illness processes. This is because the shape of the discount function underlying the medical treatment of an illness or an addiction is very important as the mathematical expression of this function can influence wrong treatment and so itself become a trans-disease factor. As such, Figure 2 can also be interpreted in the following way: if an individual suffers an addiction or an illness at a level *A*, a certain frequency *t* can reduce this level as far as *V*.

That said, under this interpretation, the function displayed in Figure 2 can better represent the progression of a certain disease with respect to the treatment (dosage or frequency) for the following reasons:

1. The decrease in the level of addiction or disease must have a lower improvement rate at the beginning, which later starts increasing. This can be best described by stating that a future discount function must verify that *V* and consequently ln *V* are convex in a neighborhood of 0. On that note, we must clarify that this paper focuses on the types of diseases whose symptoms disappear after a period of time in which a dose of medicine has been administered to the patient. Nevertheless, there are some diseases, in particular chronic ones, which are excluded from the work. A chronic disease is a persistent, or otherwise long-lasting, disease whose effects persist over a long period. A chronic course is further distinguished from a recurrent course as recurrent diseases relapse repeatedly, with periods of remission in between. However, a chronic disease may be progressive, result in complete or partial disability, or even lead to death. The expected improvement in the patient is low in the first instants of his disease but it is also logical that the curve regarding the level of the disease can decay as the number of administered doses increases. This is the particular form of the decay function we are going to analyze in this paper.
2. Following Takahashi (2006), abstinent drug addicts may be more susceptible to relapse when an abstinence period is presented as a series of divided shorter time-blocks. In terms of intertemporal choice, abstinence implies choosing larger later rewards (i.e., successful recovery from drug addiction) instead of smaller sooner ones (i.e., immediate drug intake). He points out: “My present hypothesis states that this subadditivity may result from perception of the time-interval following Weber-Fechner law. Therefore, medical and behavioral treatments which help abstinent addicts precisely perceive time-duration of abstinent period is expected to be effective.” In delay discounting, subadditivity^4^ implies that the cumulative discounted value is smaller (and the discount higher) for more subdivided intervals. For example, the discount function for 1 month will be greater than the product of the corresponding discount function values for each day. Conversely, superadditive discounting means that the discounted value is greater (and the discount smaller) when the interval is divided into subintervals.

Let us apply these concepts to our proposed discount function. If *F*(*t*) represents the concentration of pathogens in a patient when administering a treatment every *t* hours, it is possible that a dose level of reference *t*_0_ exists such that the disease can get worse if the medicine is administered every *t*_0_/2 hours and so on. This property is known as *superadditivity* which can be expressed as follows:

(2) $$F(t_{0}/2)F(t_{0}/2)>F(t_{0}).$$

In other words, the illness/addiction gets worse by partitioning the administration of tablets/drugs. Similarly, it is possible that drug administration can improve the disease if the subintervals are greater than or equal to *t*_0_. For instance,

(3) $$F(t_{0})F(t_{0})<F(2t_{0}).$$

This property is known as *subadditivity*. As we are considering behavioral and/or psychopharmacological treatment for certain diseases or addictive behavior (pathological gambling, psychiatric disorders, smoking and drugs abuse, among others), we can also represent the medicine dosage on the *x*-axis, but in the case of behavioral/psychological treatment we could represent, for example, a sequence of actions over the course of time. Similar reasoning could be performed by considering a dose of reference, *d*_0_, instead of the time of administration, such that:

- *F*(*r*)*F*(*s*) > *F*(*d*_0_), whether *r* + *s* = *d*_0_ (superadditivity).
- *F*(*r*)*F*(*s*) < *F*(*r* + *s*), provided that *r* and *s* are greater than or equal to *d*_0_ (subadditivity).

Observe that *r* and *s* can be different. This reasoning reinforces the argument whereby the function is convex in a neighborhood of 0. However hereinafter, we will focus on the dose as time frequency.

Our objective now is to find a suitable family of discount functions satisfying the two former items. This approach was introduced by Cruz Rambaud and Ventre (2014, 2015) when searching for a family of discount functions *F*(*t*) such that the following property holds:

**P**: There is a certain period of time, *t*_0_, such that the behavior of the discount function changes from subadditivity/superadditivity into superadditivity/subadditivity, i.e.:

(4) $$F(t_{1})F(t_{2})\cdots F(t_{n})<F(t),$$

if *t*_1_ + *t*_2_ + ⋯ + *t*_*n*_ = *t* ≤ *t*_0_ (which, of course, implies that all numbers *t*_*k*_ are less than *t*_0_, for *k* = 0, 1, …, *n*), and

(5) $$F(t_{1})F(t_{2})\cdots F(t_{n})>F(t),$$

if *t*_1_ + *t*_2_ + ⋯ + *t*_*n*_ = *t* and min{*t*_1_, *t*_2_, …, *t*_*n*_} > *t*_0_ (which, of course, implies that *t* and all values *t*_*k*_ are greater than *t*_0_, for *k* = 0, 1, …, *n*).

**Figure 3:**
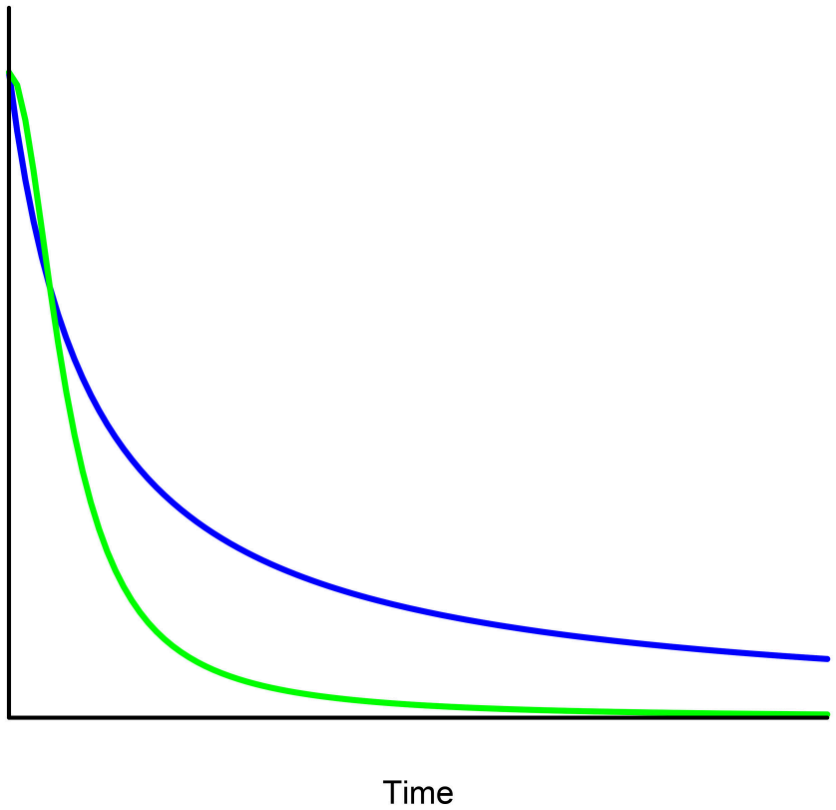
Comparison between exponentiated and simple hyperbolic discounting for the same value of parameter *i*.

As we are focusing on the disease as a delay discounting process, we will first assume that impatience as a function of delay follows the trend described in the former paragraphs. However, we are going additionally to take into account the following points:

1. If the dose must be administered every *t* hours, usually dividing in two could prove better, but this subdivision reaches a limit. In effect, there is a maximum partition for which the treatment is not efficient. In this regard, an exponentiated hyperbolic discount function (Rachlin, 2006):
  (6) $$V=\frac{A}{1+it^{k}},\;k>1$$
  or even a generalized exponentiated hyperbolic discount function:
  (7) $$V=\frac{A}{{\left(1+it^{k}\right)}^{\alpha }},\;k>1,\;\alpha >0,$$
  (see Figure 2) may be more adequate to describe an expected increasing discount rate at the beginning of the treatment, followed by a decrease in the rate. Note that *i*, *k* and α in Equations (6) and (7) represent a constant.
  Observe that function (7) is a special case of a more general *q*-exponential time discounting model (Cajueiro, 2006; Takahashi, 2007; Cruz Rambaud and Muñoz Torrecillas, 2013) with psychological time following the well-known Stevens's power law (τ = *t*^*s*^), where *q* = 0:
  (8) $$V=\frac{A}{\exp_{q}(it^{k})}=\frac{A}{{\left[1+(1-q)it^{k}\right]}^{1/(1-q)}},\;i>0,\;k>1.$$
  This model is compatible with a sharp decrease in the discount function. Indeed, Figure 3 shows a comparison of the hyperbolic discounting of monetary rewards for people with certain addictions or diseases with the exponentiated discount function representing the decrease of the disease with respect to time. Note that, for the same value of *i*, the exponentiated hyperbolic discount function (in green) is steeper than the simple hyperbola (in blue).
2. An argument which reinforces the choice of exponentiated against simple hyperbolic discounting is the so-called sequence effect as described in the experimental work by Hofmeyr et al. (2010) in the following manner: “The propensity of smokers to prefer small short-term rewards over larger delayed rewards may be mitigated, over a sequence of decisions of this kind, by encouraging or forcing them to think of the sequence as a whole.”

Focusing on a disease, let us consider a microbial population of size *N*. An *external (exposure) dose* is the amount of an agent or chemical administered to an experimental animal or human (containing this population) in a controlled experimental setting by some specific route at some specific frequency (World Health Organization, 2009). Irrespective of the *internal dose* (the external dose is that which is absorbed and enters general circulation), the dose is determined by its *frequency* and *duration*: for example, mg/kg body weight per day over a given period of time. In this context, a *dose-response curve* is a plot of the fraction of surviving organisms as a function of the exposure intensity (Peleg et al., 1997).

A *uniform effective dose* in pharmacology is the dose or amount of a drug which produces a therapeutic response or desired effect in a certain fraction of the subjects taking it. Hereinafter, we will represent the regular dose amount as *x* (in mg) and the time between two consecutive doses as *t* (in hours). Thus, the effective dose will be denoted by (*x*_0_, *t*_0_), where *x*_0_ represents the effective dose amount and *t*_0_ the time after which the drug has produced the desired effect. In some experimental studies about the possible doses for the treatment of certain diseases (for instance, Krober et al., 1990), patients were randomly selected to receive doses of *x* mg of a medicine *m* times per day, *x*/2 mg 2*m* times per day, *x*/4 mg 4*m* times, and so on. As a result, they obtained a recommended (optimal) medicine treatment. In general terms, let *x* be an amount of drug or tablets to be administered and assume that the optimal time frequency remains constant. If *F*(*y, x*) denotes the surviving population after administering *x*, *t* times per day, to an initial population of size *y*, it can be supposed that:

(9) F(y,x)=yF(1,x)=yF(x),

where *F*(1, *x*) has been denoted by *F*(*x*). As (*x*_0_, *t*_0_) is the optimal dose, one has:

(10) $$\underset{m\text{ times}}{\underset{︸}{F\left(\frac{x_{0}}{m}\right)F\left(\frac{x_{0}}{m}\right)\cdots F\left(\frac{x_{0}}{m}\right)}}>F(x_{0})$$

and

(11) $$\underset{m\text{ times}}{\underset{︸}{F(x_{0})F(x_{0})\cdots F(x_{0})}}<F(mx_{0}).$$

An alternative way of dealing with this issue could have been as follows. Essentially, let *t* be the frequency of administration (in hours) of the drug and assume that the optimal drug amount remains constant. Of course, a multiple *kt* of the frequency implies the same multiple of the amount to be administered for this time. If *F*(*y, t*) denotes the surviving population after administering an amount *x*_0_ with a frequency *t* over an initial microbial population of size *y*, it can be supposed that:

(12) F(y,t)=yF(1,t)=yF(t),

where *F*(*t*): = *F*(1, *t*) is the ratio of surviving microbes after administering *x*_0_ with a frequency *t*.

As (*x*_0_, *t*_0_) is the effective (periodic and constant) dose, one has:

(13) $$\underset{n\text{ times}}{\underset{︸}{F\left(\frac{t_{0}}{n}\right)F\left(\frac{t_{0}}{n}\right)\cdots F\left(\frac{t_{0}}{n}\right)}}>F(t_{0})$$

and

(14) $$\underset{n\text{ times}}{\underset{︸}{F(t_{0})F(t_{0})\cdots F(t_{0})}}<F(nt_{0}).$$

**Figure 4:**
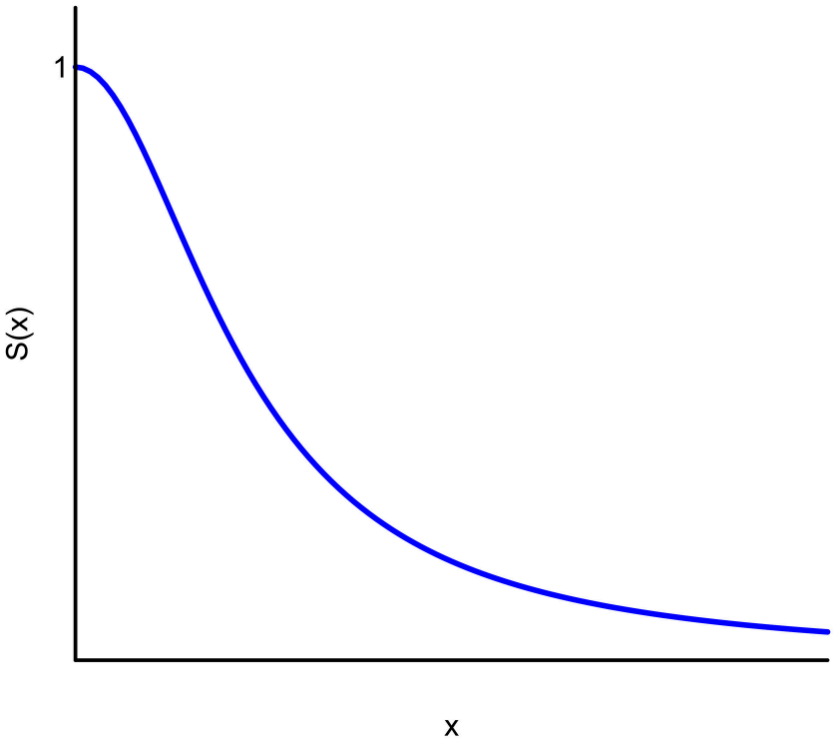
Standard dose-response curve.

By considering *F* as a function of *t*, these conditions are satisfied if *F* is superadditive to the left of *t*_0_ and subadditive to the right of *t*_0_. To be more precise, we can assume that the instantaneous discount rate of *F*, defined as:

(15) $$\delta (t)=-{\frac{\text{d }\ln F(z)}{\text{d}z}|}_{z=t}$$

increases to the left of *t*_0_ and decreases to the right of *t*_0_. An example of such a function could be (Peleg et al., 1997):

(16) $$S(x)=\frac{1}{1+\exp\{(x-x_{c})/a\}},$$

where *x*_*c*_ is a measure of the individual organism's resistance to the particular lethal agent, and *a* is an arbitrarily small numerical value. On the other hand, Altshuler (1981) proposes the following cumulative probability function representing the fraction of responders with time-response less than or equal to a time *t* when administering a dose *d*:

(17) $$P(t,d)=1-\exp\left\{-adt^{k}\right\},$$

where *a* is a constant and *k* is determined by a background response. In any case, the shape of the dose-response curve is shown in Figure 4.

Nevertheless, we are going to derive a discount function belonging to a well-known family of discount functions. To do this, we have to take into account the following result:

**Theorem** (Cruz Rambaud and Ventre, 2017). Let *F*(*t*) be a subadditive discount function and let us consider its corresponding new discount function *G*(*t*) using the time deformation *D*(*t*) = *t*^*k*^, with *k* > 1. A necessary and sufficient condition for *G*(*t*) to satisfy superadditivity and then subadditivity is that the equation:

(18) $$\frac{k-1}{kt^{k}}=\delta_{H}(t^{k}),$$

where $H\left(t\right):=\frac{\delta \left(t\right)}{\delta \left(0\right)}$, has a finite number of solutions.

As an application, the *q*-exponential discount function (Equation 8) *F*_*q*_(*t*) is subadditive for certain values of *q*. Consequently, by introducing the deformation *D*(*t*) = *t*^*k*^, *k* > 1, we can obtain a family of partially superadditive and subadditive discount functions in which we can highlight the exponentiated hyperbolic discount function (Rachlin, 2006) (Equation 6) and the generalized exponentiated hyperbolic discount function (Equation 7). Both functions are suitable for describing the progression of the level of an addiction or an illness. In summary, there are three possibilities for the variable which best describes the discounting process for the level of an addiction or illness:

1. The time frequency of the drug or therapy administration.
2. The regular amount of drug or therapy dose.
3. The delay to a certain reward that the patient can receive, once discounted, depending on the waiting time. Observe that, in this case, the discounting is applied, not to the disease or addiction, but to an original reward amount, resulting in the first row of Table 1.

## 4. Some mathematical properties of the exponentiated discount function

The so-called exponentiated discount function is not the only model to describe the progression of an addiction or disease under treatment. The advantage of this inverse S-curve time discount function is that it can be obtained from hyperbolic discounting by distorting time with a given power. It is noteworthy to highlight the relevance of Takeuchi's (2011) contribution since we need to introduce a new discount function able to describe the transition from an increasing to a decreasing instantaneous discount rate. In this section, we are going to introduce some mathematical properties which will be necessary to use the exponentiated hyperbolic discount function hereinafter. First, for the sake of simplicity, we will work with the mathematical expression of the exponentiated discount function for a $1 reward. This expression is as follows:

(19) $$F(t)=\frac{1}{1+it^{k}},\;i>0,\;k>1.$$

Let us calculate the instantaneous discount rate of this discount function:

(20) $$\delta (t):=-\frac{\text{d}\ln F(t)}{\text{d}t}=\frac{ikt^{k-1}}{1+it^{k}}.$$

Next, we question whether δ(*t*) increases or decreases. To do this, we will calculate its derivative:

(21) $$\frac{\text{d}\delta }{\text{d}t}=ik\frac{t^{k-2}(k-1-it^{k})}{{\left(1+it^{k}\right)}^{2}}.$$

Therefore, solving the equation $\frac{\text{d}\delta }{\text{d}t}=0$, we obtain the following two solutions: *t*_1_ = 0 or $t_{1}={\left(\frac{k-1}{i}\right)}^{1/k}$. Obviously, the first solution makes no sense, meaning only the second one is consistent. Moreover, observe that δ(*t*) increases in the interval ]0, *t*_1_[ and decreases in the interval ]*t*_1_, +∞[^5^. Therefore, *F*(*t*) is superadditive in the interval ]0, *t*_1_[ and subadditive in the interval ]*t*_1_, +∞[, i.e., the exponentiated discount function satisfies the property **P** defined in Section 3.

We now question whether every point (*t*_1_, *x*_1_) can be chosen as the maximum of δ(*t*). The answer is positive since (*t*_1_, *x*_1_) must satisfy the following system of equations:

(22) $$x_{1}=\frac{1}{1+it_{1}^{k}}$$

and

(23) $$t_{1}={\left(\frac{k-1}{i}\right)}^{1/k}.$$

Simple algebra shows that $k=\frac{1}{x_{1}}$ and $i=\frac{k-1}{t_{1}^{k}}$. Observe that the obtained values of both *k* and *i* are consistent because *k* > 1 and *i* > 0. Finally, using the generalized exponentiated hyperbolic discount function (7), the instantaneous discount rate is:

(24) $$\delta (t)=\frac{\alpha ikt^{k-1}}{1+it^{k}}.$$

while its derivative is:

(25) $$\frac{\text{d}\delta }{\text{d}t}=\alpha ik\frac{t^{k-2}(k-1-it^{k})}{{\left(1+it^{k}\right)}^{2}}.$$

From this, the maximum of δ(*t*) has the same abscissa as in the case of the exponentiated hyperbolic discount function, its ordinate being different. Summarizing, the (generalized) exponentiated hyperbolic discount function is a good candidate for describing the progression of illnesses or addictions with respect to their treatment as hypothesized at the beginning of this paper. In this regard, Becker and Murphy (1988) pointed out that “[p]ermanent changes in prices of addictive goods may have a modest short-run effect on the consumption of addictive goods. […] However, we show that the long-run demand for addictive goods tends to be more elastic than the demand for nonaddictive goods.” They added: “Indeed, rational persons end strong addictions only with rapid and sometimes discontinuous reductions in consumption.” As such, the dose frequency of drug administration can be adapted to the particular situation of a patient, showing the utility of equations from (22) to (25) when choosing the suitable frequency for each addict.

In the following section, we are going to show that impatience or excessive discounting as a trans-disease process can be explained by the fact that the corresponding discount rate is obtained from the hazard rate of the sum of two or more random times.

## 5. Justifying trans-disease processes

In order to explain our findings, we need the following definitions and results. A *hazard function* mathematically describes the effect that increases in waiting time have on the risk that something will happen to prevent an event from occurring (Gross and Clark, 1975). In the framework of temporal discounting, the fail represents the probability of an event occurring at *t* that will prevent the receipt of a reward, divided by the probability of the event not occurring until *t*, that is, the conditioned probability of fail. We can build a discount function based on *system reliability*: the discount function at *t* will be the reliability of the system at time *t* (denoted by *R*(*t*)), i.e., the probability that the life of the system will be greater than *t* (Cruz Rambaud and Muñoz Torrecillas, 2005):

(26) $$R(t)=1-F(t)=\exp\left\{-\int_{0}^{t}h(x)\text{d}x\right\},$$

where *F*(*t*) is the distribution function, valued at instant 0, of the random useful life of the system, and *h*(*x*) the instantaneous hazard rate at instant *x* (0 ≤ *x* ≤ *t*).

To all intents and purposes, we can consider that, at instant 0, *n* elements work and that the useful life of each component is a random variable *T*. As a consequence of the fails that happen as time passes, the number of components that still work, *N*(*t*_1_), *N*(*t*_2_), …, *N*(*t*_*n*_), decreases. Thus, we could determine the reliability and non-reliability of the system in the interval [0, *t*] as:

(27) $$R(t)=\frac{N(t)}{N(0)}\text{ and }F(t)=1-\frac{N(t)}{N(0)},$$

respectively. We can define the *instantaneous hazard rate* of a component at instant *t*, as:

(28) $$h(t)=-\underset{\Delta t\to 0}{\lim}\frac{\Delta N(t)/N(t)}{\Delta t}=-\frac{N\prime (t)}{N(t)}=-\frac{R\prime (t)}{R(t)}=-\frac{\text{d}}{\text{d}t}\ln R(t).$$

Taking into account that:

(29) $$R(t)=\int_{t}^{\infty }f(x)\text{d}x\text{ and }F(t)=\int_{0}^{t}f(x)\text{d}x$$

(*f* being the density function of *T*), one has:

(30) $$h(t)=\frac{f(t)}{1-F(t)},$$

that represents the proportion of units that fail in the interval ]*dt, t* + d*t*[ with respect to the units that continue working at time *t*.

We are going to make the hazard rate of the random variable *T*, defined in the interval [0, +∞[, equal to the instantaneous rate of a discount function (Takeuchi, 2011). The justification of our approach can be found in Cruz Rambaud and Muñoz Torrecillas (2005) in the context of systems failure. In our case, we will consider the system fail as a relapse of the substance abuser in an abstinence period.

Finally, several random variables could be considered, but for the sake of simplicity only two random variables were chosen, namely, *T*_1_ and *T*_2_ with distribution functions *F*(*t*_1_) and *F*(*t*_2_), respectively. For example, assume that *T*_1_ denotes the time in which the treatment of a smoker fails, and *T*_2_ the time corresponding to a pathological gambler. In this case, experiments can describe impatience due to the aggregation of the two addictions, *T*_1_ + *T*_2_. It is well known that the hazard rate corresponding to the sum of two (whether independent or not) random variables is greater than the hazard rate of each summand (Barlow and Proschan, 1996). Let us remind ourselves that the hazard rate is the instantaneous discount rate of the discount function describing the impatience of patients, but the treatment of the first disease needs a smaller dose. This justifies that the slope of certain discount functions can be very steep and this does not only correspond to the effect that the treatment may have in the illness since there could be a trans-disease process affecting the shape of these discount functions.

## 6. Conclusion

In this paper we have described the progression of illnesses and addictions in connection with their treatment as discount functions by considering that the “discounted disease” depends on three possible explanatory variables, viz the level of dose, the frequency of administration and the time to assess the (non)monetary rewards, all other things being the same. The level of disease is superadditive for an interval and subadditive otherwise.

**Figure 5:**
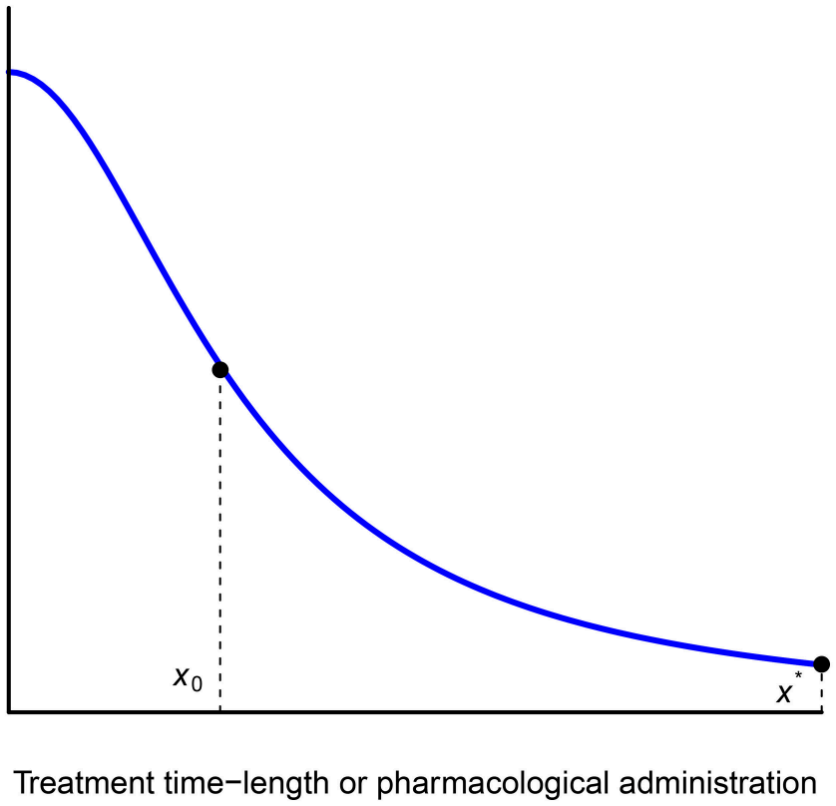
Plotting the progression of a disease.

Essentially, if x^*^ and *x*_0_ denote, respectively, the maximum and the optimal dose of medicine per day, the function representing the progression of the disease behaves as a discount function with a bounded domain [0, x^*^] where the *x*-axis now represents regular doses of the medicine or time frequencies (see Figure 5). If $n:=\left\lfloor \frac{x^{*}}{x_{0}}\right\rfloor$, then the following inequalities hold (for every integer *h* and *k* such that *k* < *n*):

$$\underset{h\text{ times}}{\underset{︸}{F\left(\frac{x_{0}}{h}\right)F\left(\frac{x_{0}}{h}\right)\cdots F\left(\frac{x_{0}}{h}\right)}}>F(x_{0})$$

and

$$\underset{k\text{ times}}{\underset{︸}{F(x_{0})F(x_{0})\cdots F(x_{0})}}<F(kx_{0}).$$

As such, despite the fact that in most experiments about illnesses and addiction processes data are fitted to hyperbolic discounting, we propose that the suitable adjustment must be to the so-called exponentiated hyperbolic discount function. This reasoning can be reinforced by the idea that most medical treatments are designed to follow a regular dosage, and this would justify the shape of the discount function. In this case, the instantaneous discount rate will be increasing up to a level of the independent variable and decreasing otherwise. An explanation of this situation is provided because in most occasions impatience is measured in patients who exhibit two or more addictions, and their impulsiveness is due to two or more diseases. Consequently, if a psychopharmacological treatment for an addiction or disease is administered based only on the impatience level shown by a patient, there could be a problem of excessive dosage. This is because impatience or excessive discounting is a trans-disease process (as indicated by Bickel et al., 2012) underlying addiction and other disorders and disease-related behavior which needs to be correctly assessed.

It should be noted that our study is a complement to the conventional economic theory of addiction (Becker and Murphy, 1988) in that our model proposes more bio-psychologically plausible characteristics for the decay of the biological influences of drug consumption after “cold turkey” (abstinence) proposed as exponential “depreciation” processes in Becker's model.

## Author contributions

All authors listed have made a substantial, direct and intellectual contribution to the work, and approved it for publication.

### Conflict of interest statement

The authors declare that the research was conducted in the absence of any commercial or financial relationships that could be construed as a potential conflict of interest.

## Footnotes

^1^For a formal definition of impatience (impulsivity), see Cruz Rambaud and Muñoz Torrecillas (2016).

^2^For example, they refer to substance abusers.

^3^Comparisons between a group with addiction and a control group.

^4^For more detailed reading on subadditive discounting, see Cruz Rambaud and Muñoz Torrecillas (2014).

^5^According to Takeuchi (2011), *F*(*t*) is an inverse S-curve.
